# Towards developing a vaccine for rheumatic heart disease

**DOI:** 10.21542/gcsp.2017.4

**Published:** 2017-03-31

**Authors:** Geethanjali Devadoss Gandhi, Navaneethakrishnan Krishnamoorthy, Ussama M. Abdel Motal, Magdi Yacoub

**Affiliations:** 1 Division of Cardiovascular Research, Sidra Medical and Research Center, Qatar Foundation, Doha, Qatar; 2 Division of Experimental Genetics, Sidra Medical and Research Center, Doha, Qatar; 3 Heart Science Centre, National Heart and Lung Institute, Imperial College London, London, United Kingdom

## Abstract

Rheumatic heart disease (RHD) is the most serious manifestations of rheumatic fever, which is caused by group A Streptococcus (GAS or *Streptococcus pyogenes*) infection. RHD is an auto immune sequelae of GAS pharyngitis, rather than the direct bacterial infection of the heart, which leads to chronic heart valve damage. Although antibiotics like penicillin are effective against GAS infection, improper medical care such as poor patient compliance, overcrowding, poverty, and repeated exposure to GAS, leads to acute rheumatic fever and RHD. Thus, efforts have been put forth towards developing a vaccine. However, a potential global vaccine is yet to be identified due to the widespread diversity of *S. pyogenes* strains and cross reactivity of streptococcal proteins with host tissues. In this review, we discuss the available vaccine targets of *S. pyogenes* and the significance of *in silico* approaches in designing a vaccine for RHD.

## Introduction

**Figure 1.**
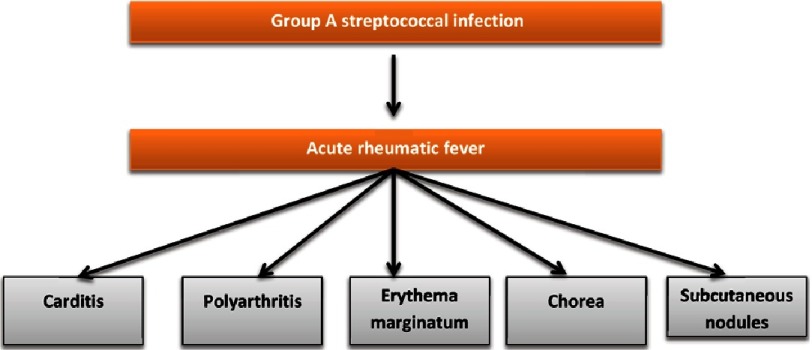
Types of inflammations caused by *S. pyogenes* infection.

Rheumatic fever (RF) is a major global burden among cardiovascular diseases and affects millions worldwide^1^. The exact pathogenic mechanism of RF is still not clear. However, it is believed that both cross-reactive antibodies and T cells have a role in this disease^2^. Acute rheumatic fever (ARF) can follow group A Streptococcus (GAS) infection in the throat, and causes inflammation of the host tissues such as joints (arthritis), central nervous system (chorea), and heart (carditis)^3,111^ (Figure 1). Severe carditis leads to chronic heart valve damage that results in rheumatic heart disease (RHD) and patients will need heart valve replacement after a period of time^4^.

It is estimated that 95% of RF and RHD occur in the developing countries and most commonly found in the regions of Australia, Pacific Islands, India, Middle East and sub-Saharan Africa^5^. GAS pharyngitis is considered to be the primary cause for RHD; however genetic polymorphism among certain molecules such as HLA class II (Human Leukocyte Antigen), TNF (Tumuor Necrosis Factor)-*α*, IL (InterLeukin)-10, IL-6 and IL-1Ra, ACE (Angiotensin I-converting enzyme) showed an increased risk of RF/RHD^5,6,112^. In addition, GAS is also responsible for a number of suppurative infections (impetigo, erysipelas, cellulitis, scarlet fever, toxic shock-like syndrome) and non-suppurative sequelae (glomerulonephritis). These suggest that the need to develop a potential vaccine against GAS infection is critical.

Various autoimmune mechanisms based on cross-reactivity between streptococcal proteins and human cardiac proteins were proposed to explain the pathogenesis of RHD^2,7,8,113^. Recently, there is an alternative hypothesis proposed based on the binding of certain rheumatogenic M serotypes such as M3 and M18 to human collagen IV results in autoantibody response to collagen which have a potential to cause acute rheumatic fever^9^. Further, it was reported that the autoantibody formed against collagen is not cross-reactive with M proteins and no molecular mimicry occurs^10^, unlike other reports around the auto immune hypothesis of RHD.

The binding of certain M serotypes to collagen IV depends on the presence of octapeptide motif called PARF motif (peptide associated with rheumatic fever) found in M and M-like proteins of *β*-hemolytic streptococcal species (group A, group C and group G streptococcus)^9^. Immunization of mice with M proteins possessing collagen binding PARF motif developed high titer of anti-collagen antibodies^9,11^. Similar anti-collagen antibodies were also observed in the sera of rheumatic fever patients, implying clinical significance of collagen binding to M proteins in the pathogenesis of RHD^9,11^.

Vaccine designing for RHD has been continuing over several decades, nevertheless, there is no protective vaccine available yet to prevent GAS infection. This might be due to several factors such as;

i) widespread diversity of *Streptococcus pyogenes* strains (more than 250 *emm* (gene encoding M protein) types)
ii) cross-reactivity between streptococcal and host proteins, and
iii) lack of relevant animal model for studying the pathogenesis of RHD^2,12–14^.

A proteomic approach, which involves the treatment of whole cells either with trypsin or Proteinase K to cleave the proteins in the outer bacterial surface and their subsequent identification by two dimensional electrophoresis (2 DE) and mass spectrophotometry, have been used to study the surface and secreted proteins of streptococcal species^15^. This approach is valuable to identify a number of cell surface associated proteins, but contamination of cell wall fractions with cytoplasmic proteins is an obstacle for using this method more widely^16^.

Recently, the proteomic approach combined with two other technologies (protein array and FACS) helps to identify well expressed, highly-conserved cell surface/secreted proteins which are considered to be important characteristics of protective antigens^17^. This combined prescreening strategy has the great advantage of reducing a large number of protein antigens undergoing animal testing in a genome-based vaccine identification method such as reverse vaccinology (RV)^17^.

The availability of genome sequences for most of the pathogens has led to the development of a new vaccine design method known as reverse vaccinology. Using this approach, researchers look at the entire genome of the pathogen to identify a novel protective antigen, instead of studying known virulent factors of the pathogen as a vaccine target. Various *in silico* tools such as Vaxign, NERVE and Rankpep have been employed for reverse vaccinology to identify a potential vaccine target for a number of pathogens^18,19^.

## Global Distribution of *emm* Types

*S. pyogenes* is a gram-positive extracellular bacterial pathogen that usually colonizes in the throat and skin thus leads to a number of suppurative and non-suppurative conditions. Several methods of classification of *S. pyogenes* strains are available, however the classification system based on M proteins (M protein serotyping) has been the most widely used method^20^. In recent years, M protein serotyping was subsequently replaced by sequencing the *emm* gene (*emm* typing) that encodes M protein^21^, and through this method more than 250 *emm* types have been identified^22^. Further *emm* sub-typing is based on the base changes within the 150-bp region encoding the predicted mature M protein N terminus relative to CDC (Centers for Disease Control and Prevention) M type reference strain^23^.

A study by Steer et al., about the distribution of *emm* types across the globe shows that there are some *emm* types (*emm1, emm12, emm4 and emm6*), which are common in high-income countries, are also prevalent in other regions such as Asia, Latin America and Middle East^24^. However, the *emm* types represented among Africa and Pacific region are distinct and the prevalence of common *emm* types found in other regions of the world were less common in these regions^23^. The reason for this difference in the molecular epidemiology of GAS disease in Africa and the Pacific might be related to the high incidence of GAS impetigo in these regions with a larger number of circulating GAS strains^23^.

Based on the available data of global *emm* type distribution^24^ it becomes evident that the *emm* types included in the 26-valent M protein based vaccine^25^ which underwent clinical trials covers most of the common *emm* types of high income countries^24^. Although, this vaccine construct would provide good coverage in Asia and Middle East with limited coverage in Africa and Pacific, a global vaccine that represents a wider spectrum of *emm* types is required for prevention of RHD worldwide. Recently, a 30-valent M protein based vaccine has shown to be effective; however it also includes only the *emm* types prevalent in North America and Europe^26^.

## Vaccine Development for RHD

**Figure 2.**
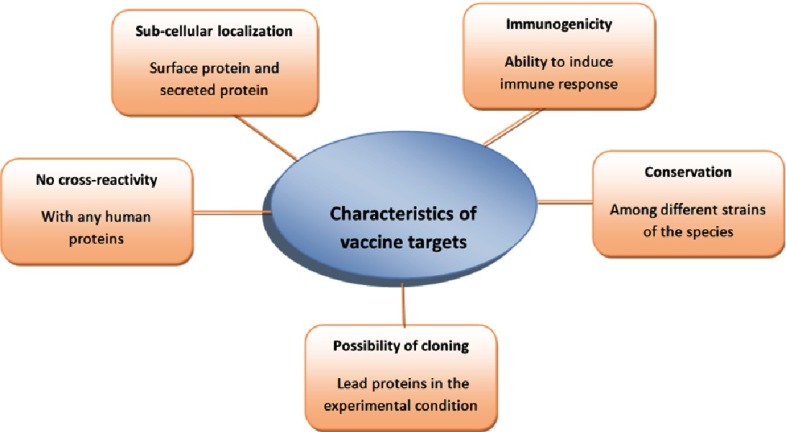
Common characteristics of a potential vaccine candidate.

Development of a vaccine for RHD started in the early 1960s with crude cell wall to purified M proteins^2^. In general, selection of vaccine candidates for any pathogen is based on few characteristics such as;

i) sub-cellular localization of the target protein,
ii) ability to induce immune responses,
iii) no molecular mimicry between target and host tissue proteins,
iv) conservation of target protein among all the available genomes of the species, and
v) possibility of cloning the target protein (e.g., proteins with no or one trans membrane helix)^18,19,27–30^ (see Figure 2).

To be a vaccine candidate, other than virulence factors, proteins with an essential role in the pathogenesis or survival can also be used as a vaccine target. For example, adhesion proteins which help in the pathogen colonization could be used for vaccine design, since the antibodies produced against this protein will prevent colonization of pathogen on the host and further progression of the disease in the host tissue^31^.

In order to develop a vaccine, surface and secreted proteins are considered to be the suitable vaccine candidates in eliciting the antibody response, but when compared to the cytosolic proteins of *S. pyogenes*, they fail to meet some of the essential characteristics that are required for development of vaccine. For instance, limited coverage of some vaccine candidates (serum opacity factor and R28) can provide protection against only a certain number of serotypes^32,33^, while other antigens (group A carbohydrate) are not effective in eliciting high concentrations of antibodies, which are required to be used as a vaccine target^32,34^. Hence, it is necessary to identify a novel vaccine candidate, which is highly immunogenic and also capable of giving protection against wide spectrum of M serotypes.

Vaccine targets for *Streptococcus pyogenes* can be classified into three major types:

i) Vaccines based on cell surface proteins.
ii) Vaccines based on secreted proteins.
iii) Vaccines based on carbohydrates.

## Vaccines Based on Cell Surface Proteins

### M protein

Among the cell surface proteins, M protein of *S. pyogenes* has been studied extensively. Especially, the hyper variable amino terminal and the highly-conserved carboxyl regions have long been the target for vaccine development against RHD due to its immunogenicity and no cross-reactivity properties^13^. However, the main limitation of using hyper variable N-terminal of M protein is the strain-specific immunity, thus multivalent vaccines have been developed by combining hyper variable N-terminal regions from different GAS serotypes.

Recently, Dale and colleagues^26^ constructed a new 30-valent M protein-based vaccine. This vaccine construct consists of N-terminal fragments (first 50 residues) of M proteins from 30 different M serotypes that are predominant in North America and Europe and further they shown to be immunogenic in rabbits. Interestingly, they have further found that this multivalent vaccine is protective against another 24 non-vaccine M serotypes, which are not included in the 30 valent vaccine construct^26^. The reason for this cross-protection may be due to the amino acid sequence similarity present in the N-terminal or high sequence similarity across the whole M protein found within the same *emm*-cluster^35^.

Although some studies suggested that the level of bactericidal antibodies produced by C-terminal region of M protein may not be adequate to give complete protection against GAS infection^32^, vaccine candidates such as Streptlncor and J14 peptide which are derived from the C-terminal of M protein shown to be protective in animal models against RHD^36,37^.

### C5a peptidase (SCPA)

SCPA (C5a peptidase of GAS) is another important cell surface molecule, which is highly conserved in all GAS serotypes and has not been associated with cross reactivity^38^. These features make it a possible vaccine candidate for GAS infection. As it is an endopeptidase, it can cleave the leukocyte binding site of the complement-derived chemotaxin C5a^39^, resulting in the inhibition of the recruitment of phagocytic cells to the site of infection, thereby helping *S. pyogenes* escape from the host immune response.

A study by O’Connor et al., started to use C5a peptidase as a vaccine component, in which they showed that measurable levels of IgA and IgG anti-SCPA antibodies are present in most of the healthy adults, but in much lower levels in uninfected children^40^. Based on this study, it was presumed that children might be susceptible to GAS infection due to the low anti-SCPA antibody response, which is necessary to prevent the colonization of GAS in the host tissues in relative to adults who have much lower incidence of the disease.

Other studies showed that the immunization of recombinant SCPA gives serotype-independent protection^41^ and prevents infection of the murine nasal mucosa-associated lymphoid tissue (NALT), which is functionally analogous to human tonsils (strong tropical site for GAS infection in humans)^38^. One advantage of using C5a peptidase as a vaccine target is that, since it shares amino acid sequence similarity with group B, C and G streptococcus, it could be used to prevent the infection caused by other groups of Streptococcus. Group C and G streptococci are known to cause infections similar to GAS, including pharyngitis and cellulitis, although they commonly cause opportunistic and nosocomial infections.

### Fibronectin (Fn) binding proteins

Fn-binding proteins of *Streptococcus pyogenes* bind to Fn present in the extracellular matrix (ECM) of host cells to interact with α5β1-integrins, which interferes with the arrangement of cytoskeleton actin, further helping the uptake of *S. pyogenes* by the host^42^.

*S. pyogenes* has 11 Fn-binding proteins, which are classified into two major types based on the presence of their binding repeats. It is found to be present in type I Fn-binding proteins, but not in type II Fn-binding proteins. Type I includes protein F1 (PrtF1)/SfbI, protein F2 (PrtF2)/PFBP, FbaA (formerly Fba), FbaB, SfbII/serum opacity factor (SOF), SfbX and Fbp54. Proteins such as M1 protein, GAPDH/Plr, Shr and Scl1 fall under type II Fn-binding proteins. Among these Fn binding proteins, studies were available for FBP54^43^, FbaA^44^, PrtF1/SFb1^45^, serum opacity factor^32^ and Shr^34^ for eliciting good immune response against GAS infection^42^.

It was reported that more than one Fn-binding protein present in some highly virulent *S. pyogenes* strains (for ex. two Fn binding proteins, *pfbp* and *prtF1*-like protein are present in the M12 strain^46^ and further the presence of Fn-binding protein is dependent on M-serotype^47^. Fn-binding proteins can also act as a genotypic marker, for example, *prtF1/sfbI* gene is commonly found in the macrolide resistant strains of Germany, Italy and Japan^48–50^ and similarly FbaB is found exclusively on bacterial surface of M3 and M18 strains which are responsible for Shock-like syndromes and not present in GAS Pharyngitis^51^. Using Fn-binding protein as a genotypic marker needs a detailed study of distribution of Fn-binding protein among different GAS serotypes worldwide.

#### i) Serum opacity factor (SOF)

SOF is a Fn binding protein expressed at the cell surface of *S.pyogenes.* It binds to Fn and fibrinogen through its conserved C-terminal domain^52^ and has the ability to opacify mammalian serum by interacting with high density lipoproteins^53^. Interestingly, it was found that the antibodies produced against opacity factor are type-specific, which can be used to determine the M serotype of GAS by using opacity factor inhibition test^2,54^.

Courtney et al., showed that SOF is able to elicit a protective immune response against SOF-positive serotypes of *S. pyogenes*^32^. They also demonstrated that antisera against one type of SOF-positive M serotype SOF2 (SOF from M serotype 2) can opsonize both homologous (M2) and heterologous SOF-positive serotypes (M4 and M28) but not SOF-negative serotype M5. This suggests that the cross-protection is possible among different SOF positive serotypes and the identification of shared epitope(s) among different SOF positive M serotypes would greatly help in combining them with other GAS vaccine candidates to develop an effective vaccine. Studies suggested that combining SOF with other protective antigens such as Fn binding protein I (SfbI) would stimulate strong systemic and mucosal immune responses which are required to prevent the disease^55^.

#### ii) Streptococcal hemoprotein receptor (Shr)

Shr is another highly conserved surface protein of GAS, which binds to hemoproteins and mediates heme acquisition^56^. In addition, it has the ability to bind with proteins of ECM such as Fn and laminin, which helps in the adherence of *S. pyogenes* to the host epithelial cells^57^. The intraperitoneal immunization of Shr elicits a strong IgG response and similarly intranasal immunization elicits both IgG and IgA response in mouse, which has proven to be protective against systemic GAS infections^34^. HtsA and SiaA are the other heme binding proteins of *S. pyogenes*^56,58^, however their role in eliciting an immune response has not yet been reported.

### *S. pyogenes* cell envelope protein (SpyCEP)

*S. pyogenes* cell envelope protein is a highly conserved, subtilin-like protease known to cleave and inactivate IL-8^59^. A study by Turner et al., shows that the immunization of mice with recombinant SpyCEP (CEP) protects against bacterial dissemination from both intramuscular soft tissue infection and intranasal upper respiratory infection caused by M81 strain^60^. In addition, they demonstrated that it could also be used to prevent the intramuscular soft tissue infection in other streptococcal species such as *S. equi*.

### R28

R28 is a highly repetitive streptococcal surface protein closely related to three different Group B Streptococcus surface proteins, α, β and Rib, which are known to be protective determinants^61–63^. R28 protein is present only in some strains of *S.pyogenes,* in particular M28, and can be used as serological marker in epidemiological studies. Although, it is considered as non-virulent in the past^64^, recent studies has suggested that it might play a role in the pathogenesis of Puerperal fever (childbed fever) based on the observation of over-representation of M28 serotype among British cases of childbed fever^65,66^. Earlier studies of R28 shows that it does not confer protective immunity^64^, however a study by Stalhammar-Carlemalm et al., showed that immunization of R28 in mouse model can elicit antibodies, which are shown to be protective against R28 expressing GAS serotypes^33^.

### Streptococcus protective antigen (Spa)

Spa is a recently identified surface protein expressed by certain strains of *S. pyogenes*^67^. A study by Dale et al., demonstrated that anti-Spa antibodies opsonize some heterologous serotypes of group A streptococci (M3 and M28) in addition to the parent strain (M18), which confirms the presence of cross-protective epitopes which could aid in the development of broad spectrum vaccines against GAS infection^67^. In contrast, another study by Ahmed et al., shows that the anti-spa antibodies raised against type 36 streptococci opsonize type 36, but fails to opsonize type 18 streptococci, indicating that opsonic epitopes of spa are type-specific^68^. There is an opening for detailed study of expression of spa protein among different strains of GAS genomes and the identification of conserved protective epitopes to use it as a universal vaccine candidate.

Another study by McLellan et al., proves that Spa is required for the virulence of type 18 streptococci along with M protein and further evidence suggests that patients with acute rheumatic fever contained antibodies against spa proteins, which clearly shows that the spa protein is highly immunogenic in humans^69^. Besides, it was found that the amino-terminal peptide fragment of Spa18 was incorporated in the recent 30 valent M protein based vaccine^26^.

### Streptococcal immunoglobulin binding protein (Sib35)

Sib35 is an immunoglobulin binding protein that binds to IgG, IgA and IgM antibodies^70^. It is an anchorless cell surface protein without LPXTG motif (cell wall anchor domain), which is also to be found partially secreted in culture supernatant^71^. Immunization of mice with Sib35 induces high IgG antibody titer, which is shown to be protective when challenging mice with GAS strains^70^.

### Vaccines based on secreted proteins

Secreted proteins of *S. pyogenes* includes at least 11 pyrogenic exotoxins such as SPEA, SPEC,SPEG, SPEH, SPEI, SPEJ, SPEK, SPEL, SPEM, streptococcal mitogenic exotoxin Z (SMEZ) and streptococcal superantigenA (SSA)^72^. Among these extracellular toxins, some are known to play an important role in causing scarlet fever, streptococcal toxic shock-like syndrome, and necrotizing fasciitis. Vaccines based on most of the secreted proteins of GAS are shown to be the most effective for systemic and invasive diseases, but have not been tested extensively for RHD^73–77^. However, a recent study by Kasper et al., shows that the immunization with toxoid SPEA is shown to be protective for nasopharyngeal infection in *S. pyogenes* MGAS8232, and this study further reveals that the mice expressing human MHC-II molecules are shown to be highly susceptible to nasopharyngeal infection^78^.

### Vaccines based on carbohydrates

Vaccines based on group A streptococcus carbohydrate (GAS CHO) are of less interest because of the cross-reactive auto-antibodies that recognize these molecules and cardiac myosin^79^. Nevertheless, a study by Sabharwal et al., shows that the active and passive immunization of GAS CHO, conjugated to tetanus toxoid, protect mice against lethal challenges with live GAS strains^80^.

They have further reported that the antibodies produced against group A carbohydrate do not show cross-reactivity with the human tissues such as brain, heart and kidney and with the cytoskeletal protein, keratin . This study was contrary to the previous findings of Shikhman et al., who demonstrated cross reactive antibodies that target group A carbohydrate and keratin^81^.

Similarly, a study by Kirvan et al., has provided evidence for the cross-reactivity between group A carbohydrate epitope, N-acetyl-beta-D-glucosamine (GlcNAc) and neuronal antigen, lysoganglioside^82^. Cunningham^83^ revealed that human monoclonal antibodies (mAbs) derived from rheumatic heart disease and Sydenham chorea share a common epitope, N-acetyl-beta-D-glucosamine (GlcNAc) of group A carbohydrate, which recognize cross-reactive structures on the heart valve and on the neuronal cells in the brain, which may lead to RHD and Sydenham chorea respectively.

### Animal models

It is not surprising that animal models for studying rheumatic heart disease is limited as human is the only host for GAS infection. Animals are not easily infected by GAS, and once infected do not maintain the disease for any extended time^2^. Lewis rat is a useful animal model for the study of immunopathogenesis of rheumatic heart disease^84^. In addition to Lewis rat, animal models such as rabbit^85^, mice^86^, pigs^87^, monkeys^88^ are employed in the study of other GAS diseases such as pharyngeal, skin and systemic infections.

## *In silico* approaches for vaccine designing against RHD

### Reverse vaccinology (RV)

Computational advancements in vaccinology considerably decrease the time scale for the discovery of novel vaccines (from 5-15 years to 2-3 years)^17,19^. RV involves analyzing the entire genome of the pathogen through *in silico* techniques to screen all the proteins encoded by the pathogen^89,110^. This aspect of studying the whole genome sequence helps to identify novel proteins which may be able to act as vaccine candidates. For example, fHbp (factor H-binding protein) of *Neisseria meningitidis* (MenB) is identified through RV and it acts as a promising antigen for the vaccine development against MenB^90,91^. Excluding MenB, RV has been successfully applied to many other pathogens including *Bacillus anthracis*^92^, *Porphyromonas gingivalis*^93^, *Chlamydia pneumonia*^94^, and *Streptococcus pneumonia*^95^, *Escherichia coli*^114^.

**Figure 3.**
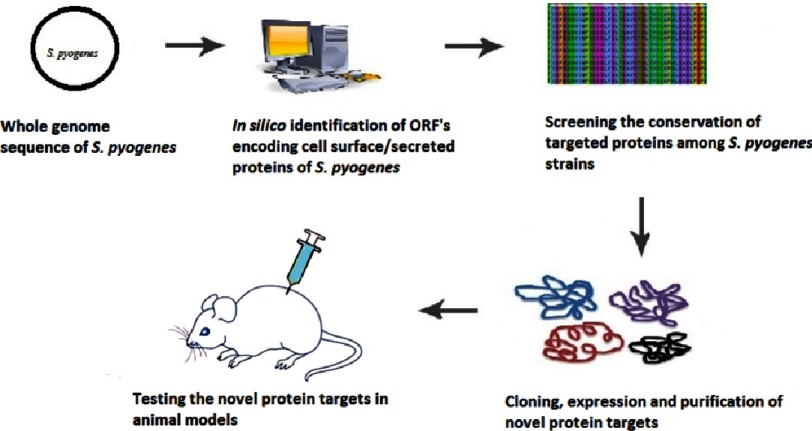
Reverse vaccinology. The procedure of reverse vaccinology starts with the study of entire genome sequence of the pathogen with the help of bioinformatics to identify all the proteins encoded by the pathogen. Novel protein targets identified through this *in silico* approach can be expressed, purified and subsequently tested for immunogenicity.

The unique advantage of *in silico* approaches using tools like PSORT, identify all the cell surface proteins encoded by the entire genome of interest (*S. pyogenes*) based on their signal sequences and other parameters^16^. Whereas, the identification of cell surface proteins by the proteomic approach face the difficulty due to contamination with cytosolic proteins during cell lysis. More than 12 genome sequences of *S. pyogenes* strains are currently available^29,96^ allowing the use of reverse vaccinology for the identification of potential vaccine targets against GAS infection, outlined in the following steps (Figure 3):

i) *In silico* identification of all the proteins encoded by the *Streptococcus pyogenes* genome.
ii) Selecting the cell surface and secreted proteins with unknown functions through the use of bioinformatics tools such as PSORTb.
iii) Screening the conservation of target proteins among different GAS serotypes and its cross-reactivity with host tissues.
iv) Cloning, expression and subsequent purification of the selected proteins and, finally,
v) testing the expressed proteins for protective immunity.

Although, RV has the limitation of identifying non-protein antigens such as polysaccharides, which are proven to be a vaccine candidate for a number of pathogens such as *Haemophilus influenza*^97^, *Salmonella typhi*^98^and *Streptococcus pneumonia*^99^, it is helpful to identify the proteins that may fail to express *in vitro* conditions. Since, the pathogenesis of *Streptococcus pyogenes* is is poorly understood, identification of unknown proteins through RV could help in understanding the mechanism of GAS pathogenesis. Spy0416^15^ andSpy1325^100^ are two novel antigens which are identified by studying the genome sequences of *S. pyogenes*.

**Figure 4.**
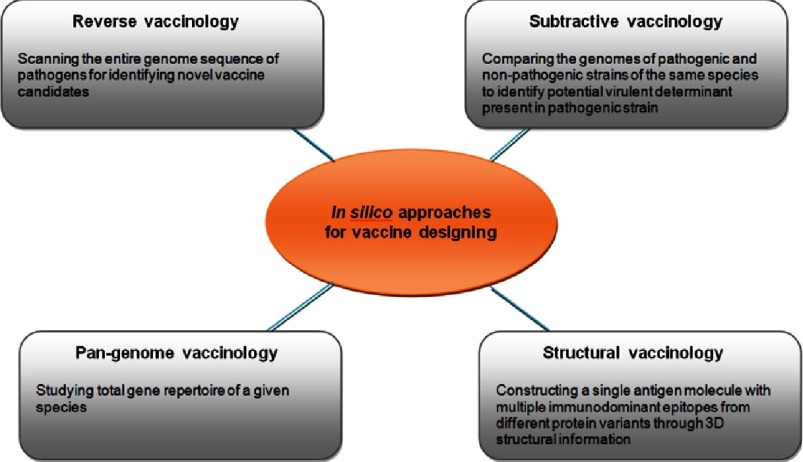
Different types of *in silico* approaches for identifying potential targets for vaccine development.

Various advances have been made to the classical RV approach, such as subtractive RV and pan-genome reverse vaccinology (Figure 4). The bacterial pan-genome concept was proposed by Tettelin et al.,^101^ when studying the genome sequences of eight different isolates of *S. agalactiae,* which represents the total genetic diversity of the species.

Pan-genome represents all the genes present in different isolates/strains of the single species. In general, this method classifies the genome into three parts; i) core-genome (set of genes present in all strains/isolates), ii) dispensable genome (genes present in some but not in all the strains/isolates), and iii) strain-specific genes (genes present only in the particular strain/isolate)^29^. Though the core gene present in all strains seems to be a potential vaccine candidate, they are likely to be less immunologic in any pathogen^29^.

In contrast, dispensable genes – present only in a subgroup of strains – might be an invaluable source of novel antigens, which could encode virulence factors^102^. A pan-genome based vaccine designed for the first time for *S.agalactiae* (GBS), in which the final vaccine consist of four antigens derived from core and dispensable genomes of eight isolates of GBS, provides universal strain coverage and gives similar type of protection when using capsular carbohydrate vaccine of GBS^103^. The pan-genome based approach has also been used to identify the pilus antigen (Lancefield T antigen) of GAS through the analysis of five GAS genomes, which are shown to confer protective immune response in mice^104^. The pan-genome approach was further extended to design vaccines based on 3-dimensional structural information, referred as structural vaccinology^115,116^.

### Structural vaccinology

Structural vaccinology utilizes 3D structural data of target antigens/proteins, and has been successfully applied to study the factor H-binding protein (fHbp, GNA1870) of *N. meningitidis*^91^. It refers to the engineering of multiple immunodominant conformational epitopes (discontinuous epitopes) from different variants (proteins) into a single molecule to give a universal protection^31^. fHbp is a surface lipoprotein identified recently by studying the MenB genome sequence^90^. fHbp is known to be highly immunogenic, however it has more than 500 known variants, which are broadly classified into variant groups 1, 2 and 3^105^. There is no cross-protection observed among the three different groups of variants and a single antigen that covers all the three groups of variants of fHBp would be considered a novel vaccine.

From the 3D structural information of fHbp, determined through nuclear magnetic resonance (NMR)^106^, it is clear that the conformational epitopes of variant 1, 2 and 3 are located in non-overlapping regions. This data leads to designing a fHbp chimeric protein with variant 1 as a scaffold to carry patches of amino acids from the surface of variants 2 and 3 to induce a broad spectrum of immune response^91^.

Structure-based vaccine design has also been employed to study group B streptococcus pilus antigen^107^, influenza virus HA antigen^108^, and HIV gp120^109^. Recent functional classification of M protein variants into 48 *emm*-clusters^37^ gives a new insight that there might be a possibility of common epitopes existing among different M serotypes. In this instance, structural vaccinology would be helpful to engineer the common epitopes from different M protein variants into a single molecule which could show universal protection against all available M serotypes.

## Conclusion

Recent 30-valent vaccines based on M protein, which are shown to be protective against RHD in the US and European population^26^, are undergoing clinical trials. A global vaccine that covers other regions of the world, especially low-income countries, would be extremely helpful to eradicate RHD completely. With the recent computational advancements in the field of vaccinology^110^, we hope that a protective vaccine against RHD is within reach, either through identification of novel antigens by RV, or through structure-based design of known antigens of GAS.
